# Mepolizumab reduces healthcare resource utilization in patients with chronic rhinosinusitis with nasal polyps: a linked EMR and claims-based pre-post study

**DOI:** 10.1186/s13223-026-01038-w

**Published:** 2026-06-21

**Authors:** Anna Swenson, Waseem Ahmed, Jared Silver, Shiyuan Zhang, Amy Edgecomb, Lingjiao Zhang, Rafael Alfonso-Cristancho, Richard Gliklich

**Affiliations:** 1 OM1, Inc. Boston, Boston, MA USA; 2 Global Data Generation, GSK, London, UK; 3 US Medical Affairs − Respiratory, GSK, Durham, NC USA; 4 Global Real-World Evidence and Health Outcomes Research, GSK, Collegeville, PA USA; 5 Anti-Infectives and Respiratory, US Real-World Evidence and Health Outcomes Research, GSK, Philadelphia, PA USA; 6 Biostatistics, GSK, Collegeville, PA USA; 7 Harvard Medical School, Boston, MA USA

**Keywords:** Endoscopic sinus surgery, Corticosteroid use, Chronic rhinosinusitis, Health care economics

## Abstract

**Background:**

Real-world evidence on the effectiveness of mepolizumab at reducing healthcare resource utilization (HCRU) and clinical symptoms in patients with chronic rhinosinusitis with nasal polyps (CRSwNP) is limited.

**Methods:**

This real-world study used linked electronic medical record and claims data from the OM1 Real-World Data Cloud of clinician networks in the US, including an ear, nose and throat physician registry. CRSwNP-related HCRU, procedures, concomitant medications, and sign and symptom outcomes were assessed in adult patients with CRSwNP who initiated mepolizumab on/after July 29, 2021 (index date), with ≥1 mepolizumab record, data available for ≥12 months pre- and ≥6 months post-index (follow-up/post-mepolizumab period).

**Results:**

There was a significant difference in CRSwNP-related HCRU 6-months post- versus pre-mepolizumab initiation (*N* = 245), mean difference in outpatient visits (95% confidence interval): −0.82(−1.10, −0.53), *p* < 0.0001; otolaryngologist visits: −0.35(−0.54, −0.16), *p* = 0.0004; allergist visits: −0.28(−0.43, −0.14), *p* = 0.0001. Similar reductions were observed for all-cause HCRU. At 6-months post-mepolizumab, proportion of patients with oral corticosteroid and oral antibiotic prescriptions substantially reduced (36% pre- vs. 27% post-; and 38% pre- vs. 23% post-, respectively). There were clinically meaningful improvements in symptoms recorded 6-months post- versus pre-mepolizumab, with fewer patients experiencing anosmia (−53%), facial pain/pressure (−35%), nasal discharge (−32%) and nasal obstruction (−29%).

**Conclusions:**

In real-world practice, mepolizumab is associated with reduced HCRU and improved clinical symptoms, potentially alleviating medication burden in patients with CRSwNP. These results indicate, beyond improvements shown in randomized controlled trials, mepolizumab may reduce HCRU disease burden as early as 6-months post-initiation. This study offers real-world insight that may support clinical decision-making with mepolizumab.

**Supplementary Information:**

The online version contains supplementary material available at https://doi.org/10.1186/s13223-026-01038-w.

## Introduction

Type 2 inflammation is implicated in the pathogenesis of several respiratory diseases [1, 2], including chronic rhinosinusitis with nasal polyps (CRSwNP) [3]. Mepolizumab is a first-in-class eumamonoclonal antibody thpolizat specifically targets interleukin-5, a key driver of type 2 inflammation [4]. The efficacy of mepolizumab for patients with CRSwNP was established in the SYNAPSE trial (NCT03085797) [5]. The Phase III, double-blind, placebo-controlled trial demonstrated mepolizumab to significantly reduce polyp size, the occurrence of nasal surgeries, systemic corticosteroid (SCS) use, and improve nasal symptoms and health-related quality of life. These findings were pivotal in the approval of mepolizumab as an add-on treatment to the standard of care for patients with CRSwNP [4, 5].

While there is real-world evidence (RWE) of mepolizumab’s effectiveness in patients with severe asthma and comorbid CRSwNP using claims-based databases [6, 7], most published RWE studies relying on clinical data have been small and focused on European populations [8–12]. However, RWE for United States (US) patients with CRSwNP remains limited [13, 14]. Larger studies have primarily been claims-based, lacking detailed correlations between patient symptoms and prescriptions. This study addresses these gaps by combining claims data with medical records to offer a more comprehensive view of healthcare resource utilization (HCRU) in a relatively larger and more representative sample of US-based patients [15, 16].

To bridge this data gap, this study explored the effectiveness of mepolizumab for CRSwNP in a real-world setting, aiming to elucidate HCRU and provide further insights into clinical symptoms using a large routine practice registry. The OM1 Real-World Data Cloud (RWDC) links relevant specialist medical records (electronic medical records [EMR]) and claims data from various clinician networks across the US, including nasal specialist registries. This extensive, linked registry enables a comprehensive evaluation of mepolizumab’s impact on CRSwNP-related outcomes in a clinical setting. In contrast, databases relying solely on claims data are limited by their inability to capture detailed patient information.

This study evaluated CRSwNP-related HCRU, diagnostic and surgical procedures, concomitant medications and sinonasal symptoms to compare endpoints pre- and post-mepolizumab initiation, in a cohort of patients with CRSwNP treated in a real-world setting.

## Methods

### Study design

This was a retrospective cohort study (GSK ID: 217530) of de-identified EMR and claims data using the OM1 RWDC. The OM1 RWDC is a regularly updated, multi-source dataset derived from EMR that links relevant specialist medical record and claims data from a range of clinician networks in the US, including the American Academy of Otolaryngology-Head and Neck Surgery Foundation Reg-ENT^SM^ Clinical Data Registry. As this study was a retrospective analysis of de-identified data, informed consent or ethics approval were not required. The database included data from 15,664 patients who initiated mepolizumab on or after July 29, 2021 (date mepolizumab received US Food and Drug Administration approval for use in patients with CRSwNP). Study outcomes were assessed using a pre- and post-mepolizumab initiation study design (Supplementary Fig. 1).

### Patient population

Patients initiated mepolizumab on or after July 29, 2021 (index date) and the identification period ran until December 31, 2022, patients were ≥ 18 years of age on the index date, with ≥ 1 mepolizumab record, both EMR and claims data available for ≥ 12 months pre- and ≥ 6 months post-index date, with a specialist EMR encounter during the 12-month baseline period, and ≥ 1 CRS-associated NP diagnosis code during the 12-month baseline period or on the index date. There were two pre-mepolizumab periods, (1) defined as 12-months pre-index and (2) defined as 6-months pre-index.

The post-mepolizumab period was the 6-month follow-up period post-index date (Supplementary Fig. 1).

### Outcomes

Patient baseline demographics and characteristics were assessed during the 12-month baseline period (including the index date). Study outcomes were evaluated pre- and post-mepolizumab initiation for both the main and sensitivity analyses.

The primary objective was to evaluate CRSwNP-related HCRU. CRSwNP-related HCRU was assessed by the proportion (n [%]) of outpatient, specialist and emergency department (ED) visits 6- and 12-months pre- and 6-months post-mepolizumab initiation. Additionally, changes in CRSwNP-related HCRU were assessed by mean difference (95% confidence interval [CI]) in the number of outpatient, specialist and ED visits 6-months pre- versus 6-months post-mepolizumab initiation. All visits, including those associated with mepolizumab administration were included in the assessment of HCRU. The secondary objectives were to evaluate CRSwNP-related diagnostic and surgical procedures, concomitant medications, signs and symptoms; asthma-related medication use and all-cause HCRU. The proportion (n [%]) and type of CRSwNP-related diagnostic and surgical procedures, medication records, signs and symptom mentions and asthma-related medication prescription records were assessed 6- and 12-months pre- and 6-months post-mepolizumab initiation; mean difference (95% CI) in the number of diagnostic and surgical procedures, medication records, signs and symptom mentions and asthma-related medication prescription records were also assessed 6-months pre- versus 6-months post-mepolizumab initiation.

CRSwNP-related signs and symptoms were assessed by the number of mentions recorded on distinct days and were previously extracted from clinical notes from the OM1 data. All-cause HCRU was assessed by proportion (n [%]) of outpatient, specialist and ED visits 6- and 12-months pre- and 6-months post-mepolizumab initiation. Mean differences were calculated between 6-months pre- and 6-months post-mepolizumab periods for each patient, based on paired data.

### Statistical methods

A minimum sample size of 174 patients was required to achieve 80% power to detect a mean difference of 0.5 visits at a two-sided 5% level of significance.

Continuous variables were summarized by mean (standard deviation [SD]) and median (first quartile and third quartile). Categorical variables were summarized by count and percentage for each category. The denominator for the percentages was the number of patients with non-missing data; missing data were not imputed. Both p-values and 95% CI were produced for the pre-/post-mepolizumab analyses. Analysis datasets were prepared by Structured Query Language, and the analysis output was produced using SAS Software (SAS Institute Inc., Cary, NC, USA), version 9.4.

A sensitivity analysis of patients with ≥ 2 mepolizumab records during the 6-month follow-up period was conducted to counter the bias potentially introduced by patients who did not continue their mepolizumab treatment; this analysis assessed CRSwNP-related HCRU outcomes only. Additionally, a sensitivity analysis in a patient population without severe asthma was conducted to evaluate outcomes in patients solely affected by CRSwNP, to evaluate whether changes with mepolizumab were due to improvements in CRSwNP and not secondary to improved severe asthma control, this assessed both CRSwNP-related HCRU and concomitant medication outcomes. To be included in the asthma-related medication sensitivity analysis, patients required ≥ 1 International Classification of Diseases, Tenth Revision, Clinical Modification J45.5X diagnosis code for severe asthma during the 12-month baseline period. Patients were not excluded due to previous biologic use.

## Results

### Patient population

Of the 15,664 patients who initiated mepolizumab during the study period, 245 patients met the eligibility criteria and were included in the overall population (Supplementary Fig. 2). The mean age (SD) of patients was 57.6 (14.6) years, a near 1:1 ratio of female to male patients were included (124/245, 51%: 121/245, 49%) and most were White (170/245, 69%). The most common healthcare insurance options for patients included commercial (91/245, 37%) or Medicare (59/245, 24%). The most frequent comorbidities in the patient population were allergic rhinitis (168/245, 69%) and asthma (159/245, 65%), nearly a third of patients suffered from severe asthma (76/245, 31%). (Table 1).

**Table 1:** Baseline demographics and clinical characteristics

Characteristics	Overall population* N = 245
*Age (years)*	
Mean (SD)	57.6 (14.6)
*Sex*,* n (%)*	
Female	124 (51)
*Race*,* n (%)*	
White	170 (69)
Black or African American	30 (12)
Asian	10 (4)
Other	13 (5)
Unknown or not reported	22 (9)
*Ethnicity*,* n (%)*	
Non-Hispanic or Latino	184 (75)
Hispanic or Latino	20 (8)
Unknown or not reported	41 (17)
*Insurance type*,* n (%)*	
Commercial	91 (37)
Medicare	59 (24)
Medicaid	5 (2)
Other	7 (3)
Unknown	83 (34)
*Charlson comorbidity index*	
Mean (SD)	1.99 (1.98)
*Charlson comorbidity index category*,* n (%)*	
0–1	143 (58)
2–3	68 (28)
4–5	19 (8)
≥ 6	15 (6)
*Comorbidities*,* n (%)*	
Allergic rhinitis	168 (69)
Asthma (including severe asthma)	159 (65)
Severe asthma	76 (31)
Acute sinusitis	53 (22)
COPD	35 (14)
Sleep apnea	32 (13)
*CRSwNP-related procedures*,* n (%)*	
Sinus debridement	43 (18)
ESS	36 (15)
Nasal endoscopy	182 (74)
Sinus CT	66 (27)
Polypectomy	4 (2)
*CRSwNP-related concomitant medications*	
OCS	124 (51)
Oral antibiotics	121 (49)
INCS^†^	79 (32)
*CRSwNP-related signs and symptoms*, *n (%)*	
Nasal obstruction	122 (50)
Nasal discharge	106 (43)
Facial pain/pressure	57 (23)
Anosmia	46 (19)

	Asthma subgroup (*n* = 76)
*Asthma-related medication* ^‡^	
LABA	0 (0)
Theophylline	0 (0)
Cromolyn	0 (0)
Oral albuterol	0 (0)
Inhaled SABA	24 (32)
Leukotriene modifiers	21 (28)
ICS	18 (24)
ICS/LABA	18 (24)
ICS/LABA/LAMA	13 (17)

*At 12 months pre-mepolizumab initiation; ^†^INCS refers to prescriptions only and does not include over-the-counter medications; ^‡^At 6 months pre-mepolizumab initiation

COPD, chronic obstructive pulmonary disease; CRSwNP, chronic rhinosinusitis with nasal polyps; CT, computed tomography; ESS, endoscopic sinus surgery; ICS, inhaled corticosteroid; INCS, intranasal corticosteroid; LABA, long-acting β_2_-agonist; LAMA, long-acting muscarinic antagonist; OCS, oral corticosteroid; SABA, short-acting β_2_-agonist; SD, standard deviation

During the 12-month baseline period, few patients underwent sinus debridement (43/245, 18%), endoscopic sinus surgery (36/245, 15%), or polypectomy (4/245, 2%), whereas nasal endoscopy (182/245, 74%) and sinus computed tomography (CT; 66/245, 27%) were more common (Table 1). CRSwNP-related concomitant medications at baseline included oral corticosteroids (OCS; 124/245, 51%), oral antibiotics (121/245, 49%), and intranasal corticosteroids (INCS; 79/245, 32%). Some patients had received other biologics indicated for CRSwNP during the 12-month baseline period, most frequently dupilumab (33/245, 13%), followed by omalizumab (9/245, 4%) and benralizumab (8/245, 3%). Baseline symptom documentation included nasal obstruction (122/245, 50%), nasal discharge (106/245, 43%), facial pain/pressure (57/245, 23%), and anosmia (46/245, 19%) (Table 1).

### CRSwNP-related HCRU

**Fig. 1:**
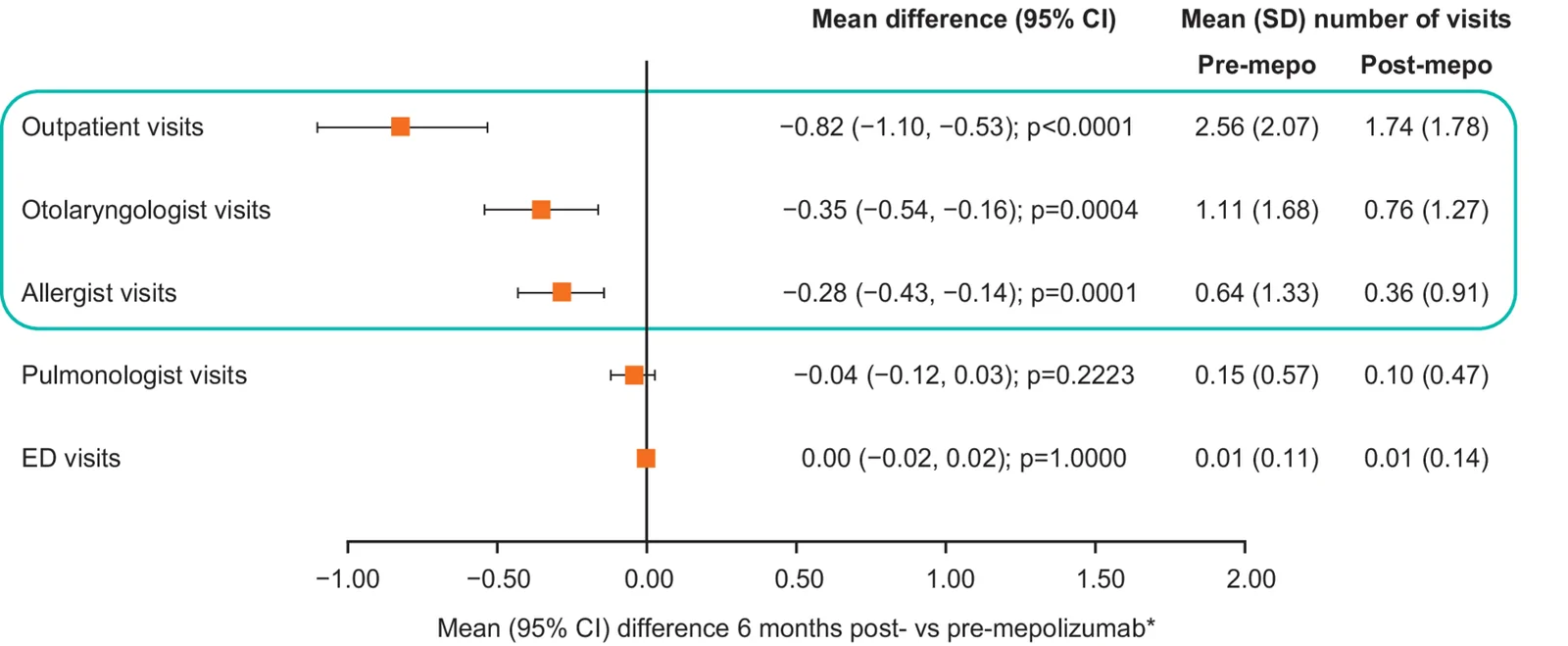
CRSwNP-related HCRU, including visits associated with mepolizumab administration pre- versus post-mepolizumab initiation.*Changes in CRSwNP-related HCRU were assessed by mean difference (95% CI) in the number of outpatient, specialist and ED visits 6-months pre- versus 6-months post-mepolizumab initiation. CI, confidence interval; CRSwNP, chronic rhinosinusitis with nasal polyps; ED, emergency department; HCRU, healthcare resource utilization; mepo, mepolizumab; SD, standard deviation

At baseline, outpatient and otolaryngologist visits were more common than allergist or pulmonologist visits, and few visits were made to the ED (Supplementary Table 1). The mean number of CRSwNP-related outpatient, otolaryngologist and allergist visits, including visits associated with mepolizumab administration, were significantly reduced 6-months post- versus pre-mepolizumab initiation, *p* < 0.0005 (Fig. 1). For CRSwNP-related outpatient visits (mean difference [95% CI] −0.82 [−1.10, −0.53], *p* < 0.0001), this equates to nearly one fewer visit per 6-month period post-mepolizumab (or a 32% reduction). While not significant, there was a trend for a reduction in pulmonologist visits post-mepolizumab (Fig. 1).

### CRSwNP-related procedures, concomitant medications, and signs and symptoms

**Fig. 2:**
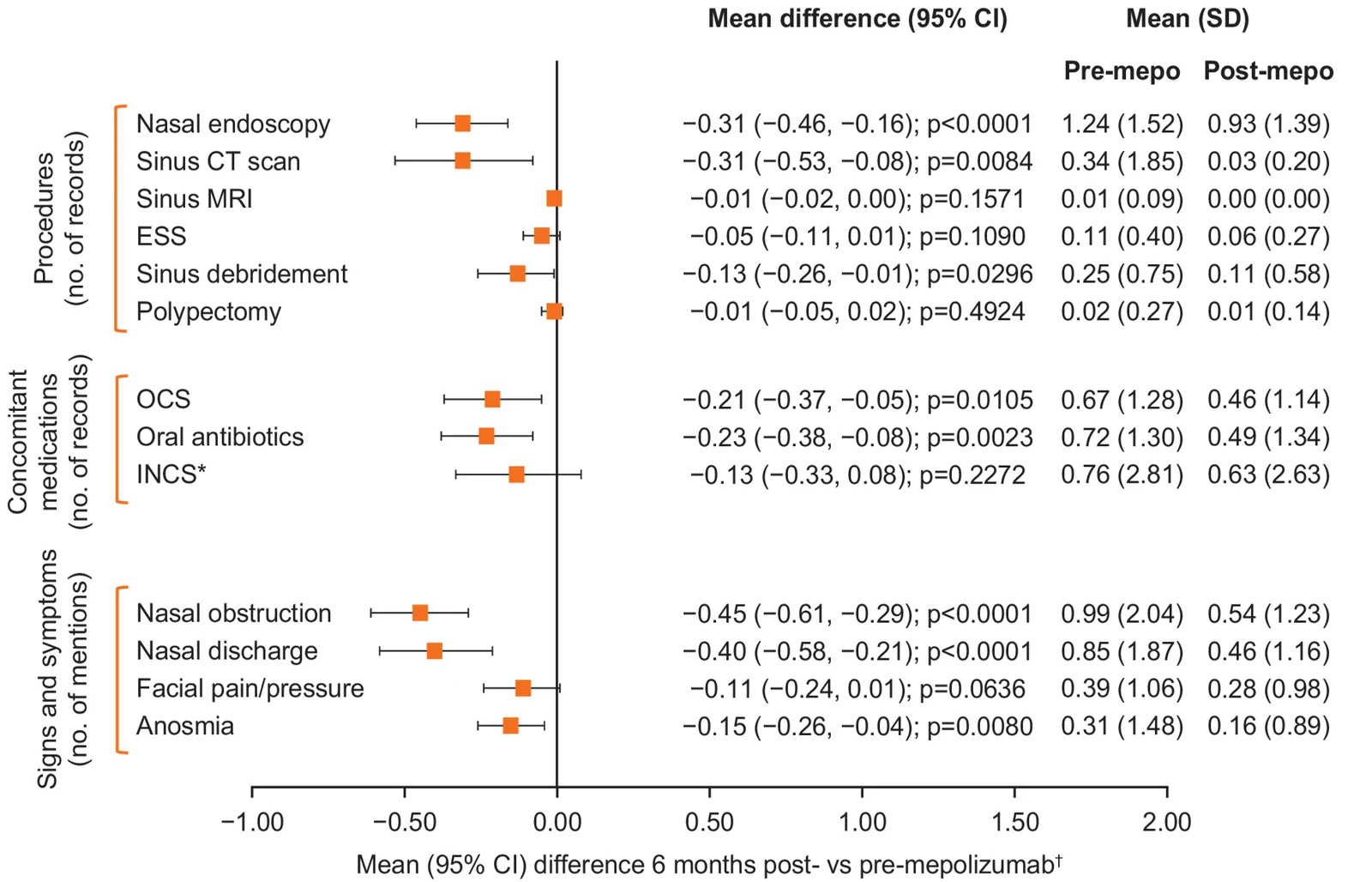
CRSwNP-related procedures, concomitant medications and signs and symptoms pre- versus post-mepolizumab initiation.*INCS refers to prescriptions only and does not include over-the-counter medications; ^†^Changes in clinical outcomes were assessed by mean difference (95% CI) in the number of diagnostic and surgical procedures, medication records, signs and symptom mentions and asthma-related medication prescription records 6-months pre- versus 6-months post-mepolizumab initiation. CI, confidence interval; CRSwNP, chronic rhinosinusitis with nasal polyps; CT, computed tomography; ESS, endoscopic sinus surgery; INCS, intranasal corticosteroid; mepo, mepolizumab; MRI, magnetic resonance imaging; OCS, oral corticosteroid; SD, standard deviation

The most notable improvements in CRSwNP-related procedures were in the mean number of nasal endoscopy and sinus CT scans, each of which significantly reduced in the 6-months post- versus pre-mepolizumab initiation (Fig. 2); the proportion of patients with procedures reduced by 64% and 44%, respectively (Supplementary Table 1). The proportion of patients requiring sinus debridement and endoscopic sinus surgery also decreased 6-months post- versus pre-mepolizumab initiation (Supplementary Table 1), and changes in the mean number of debridement, polypectomy, or other sinus procedures were minor and non-significant (Fig. 2).

There was a 25–39% reduction in the proportion of patients with CRSwNP-related medication prescriptions, fills or administrations (Supplementary Table 1) for OCS, oral antibiotics, and INCS post- versus pre-mepolizumab initiation, although the mean change in number of medication records was not significant for INCS (Fig. 2). This did not take into account possible over-the-counter INCS use, as INCS records in this dataset reflect prescription activity only.

Clinically meaningful improvements were seen across all sign and symptom outcomes. There was a significant reduction in nasal obstruction, nasal discharge and anosmia mentions recorded post- versus pre-mepolizumab initiation (*p* < 0.05, Fig. 2). There was a 53% reduction in the proportion of patients experiencing anosmia post-mepolizumab; as well as an approximately 30% reduction in nasal discharge and nasal obstruction (Supplementary Table 1). There was also a 35% reduction in the proportion of patients experiencing facial pain/pressure post-mepolizumab (Supplementary Table 1) (the mean difference in facial pain/pressure mentions recorded was non-significant [95% CI] −0.11 (−0.24, 0.01), *p* = 0.0636) (Fig. 2).

### Asthma-related medication use in patients with severe asthma

In those with comorbid severe asthma, (*n* = 76), from baseline until 6-months post-mepolizumab, 32% of patients were receiving inhaled short-acting β_2_-agonists (SABA), and 28% were on leukotriene modifiers. Additionally, 24% of patients were prescribed inhaled corticosteroids (ICS) and ICS/long-acting β_2_-agonist (LABA) combinations. Notably, 17% of patients were receiving ICS/LABA/long-acting muscarinic antagonist (LAMA) triple therapy (Table 1). Non-significant reductions in medication use were observed 6-months post- versus pre-mepolizumab initiation; the mean difference in number of ICS records was −0.16, 95% CI −0.36, 0.04, *p* = 0.1221; −0.32 for inhaled SABA, 95% CI −0.63, 0, *p* = 0.0478; −0.05 for ICS/LABA, 95% CI −0.30, 0.20, *p* = 0.6762; and −0.22 for ICS/LABA/LAMA triple therapy, 95% CI −0.45, 0.00, *p* = 0.0490, respectively.

### All-cause HCRU

Broadly similar reductions were observed for all-cause HCRU compared with the overall population. There was a 9%, 19% and 29% reduction in outpatient, otolaryngologist and allergist visits post- versus pre-mepolizumab initiation, respectively.

### Sensitivity analysis: patients with ≥ 2 mepolizumab records

Of the overall population, 158/245 (64.5%) patients had ≥ 2 mepolizumab records. Baseline characteristics were broadly similar to the overall population; the mean age of patients (SD) was 57.6 (14.8) years, and the majority had commercial (64/158, 41%) or Medicare (37/158, 23%) insurance coverage.

Similar to the overall population, a significant difference was observed in the mean number of CRSwNP-related outpatient, otolaryngologist, and allergist visits, including visits associated with mepolizumab administration (Supplementary Fig. 3). Non-significant changes were observed in the mean number of pulmonologist or ED visits that were similar to the overall population (Supplementary Fig. 3).

### Sensitivity analysis: patients with CRSwNP without comorbid severe asthma

During the 12-month baseline period, 169/245 patients had CRSwNP without comorbid severe asthma, of which the mean age was (SD) of 56.3 (15.1) years; most had commercial insurance (65/169, 38%) and 20% of patients were covered by Medicare insurance (34/169).

The mean difference in the number of CRSwNP-related outpatient visits, otolaryngologist visits, and allergist visits were similar to those in the overall population, post-mepolizumab initiation (Supplementary Fig. 4).

The significant mean difference observed in CRSwNP-related nasal obstruction and nasal discharge mentions recorded were also similar to those in the overall population, post-mepolizumab initiation (Supplementary Fig. 4).

The mean difference in OCS and oral antibiotic prescriptions, fills or administrations were numerically higher than those in the overall population, post-mepolizumab initiation (Supplementary Fig. 4).

## Discussion

This study demonstrates that in a real-world setting, mepolizumab is associated with reductions in HCRU as early as 6-months post-initiation, with concurrent improvements in clinical symptoms, and may help alleviate medication burden in patients with CRSwNP. This reaffirms observations from the SYNAPSE trial, but in a broader patient population, where mepolizumab significantly reduced the need for NP surgery, improved key symptoms, including sense of smell and nasal discharge, and reduced the use of SCS [5].

In the overall population, a statistically significant difference was observed in CRSwNP-related HCRU, including total outpatient, otolaryngologist, and allergist visits post-mepolizumab initiation; notably, these measures also factor in visits associated with mepolizumab administration, substantially strengthening the findings of this study. It is important that healthcare visits associated with mepolizumab use are accounted for when assessing HCRU to help determine whether the burden of a medication type (i.e., the additional healthcare visits associated with the medication) outweighs the medication benefit (associated clinical improvement).

As this is a registry-based study, a sensitivity analysis was conducted to confirm whether the effect of mepolizumab observed in the overall population is also seen in patients with repeated mepolizumab administrations, a common and crucial means of treatment for many patients managing chronic inflammatory conditions. In this population of patients with ≥ 2 mepolizumab records, significant differences in HCRU similar to the overall population were also observed 6-months post-mepolizumab, including total outpatient, otolaryngologist, and allergist visits; demonstrating that in a clinical setting, the effects of mepolizumab are consistent in patients with one and more than one mepolizumab administration.

Given the high prevalence of severe asthma in the cohort, it was necessary to address whether the benefits observed with mepolizumab were attributed to improvements in CRSwNP rather than asthma-related effects, especially as REALITI-A showed a marked reduction in HCRU among patients with severe asthma treated with mepolizumab [17]. Therefore, another sensitivity analysis was conducted in patients without comorbid severe asthma, because patients may have been prescribed mepolizumab for asthma, rather than CRSwNP. This analysis demonstrated similar reductions in CRSwNP-related HCRU, and greater reductions were seen in the number of concomitant medications versus the overall population, indicating that improvements were likely CRSwNP-related and not necessarily secondary to asthma symptom control with mepolizumab.

In the overall population, statistically significant decreases in CRSwNP-related diagnostic (nasal endoscopy and sinus CT) and surgical procedures (sinus debridement), CRSwNP-related medications (OCS and antibiotic), and sino-nasal symptoms (nasal obstruction, nasal discharge, anosmia) were also observed 6-months post-mepolizumab initiation, compared with the pre-mepolizumab period. Despite the report of fewer sinus CT scans versus the pre-mepolizumab period, it should be noted that repeat CT scans after treatment initiation may not be standard practice unless there are concerns for subsequent intervention needs, or complications previously undetected before treatment initiation. Therefore, the reduction in CT scans may not be reflective of improved clinical condition and rather standard practice. Whereas, due to the established effectiveness of mepolizumab at reducing severe asthma-related medication use [18, 19], the trend toward reductions in asthma-related medication use in the overall population is expected and is in alignment with previous findings. Additionally, these findings substantiate the improvements in signs and symptoms reported in the SYNAPSE trial (treatment effect in composite visual analog scale score [nasal obstruction, nasal discharge, throat mucus and loss of smell]: −2.68: 95% CI −3.44, −1.91, *p*=0.020) [5] in real-world clinical practice.

This study assessed outcomes 6-months post-mepolizumab initiation; this is a clinically relevant time period aligning with the most recent European Forum for Research and Education in Allergy and Airway (EUFOREA) treatment guidelines, which recommend assessing biologic response at 6 months [20]. A post hoc analysis of the SYNAPSE trial has already found that clinical improvements achieved with mepolizumab increased progressively over time (from Week 24 to Week 52) i.e., are maintained beyond the 6-month follow-up period applied here [21], indicating that the treatment benefits reported here have the potential to be sustained over time.

A recent retrospective claims-based study of mepolizumab in patients with asthma and comorbid CRSwNP provides complementary findings to this study. The inclusion criteria were broadly similar to this protocol; however, unlike this study, patients were required to have ≥2 mepolizumab claims during the post-index period, with no mepolizumab use pre-index, and no use of benralizumab, omalizumab, reslizumab, or dupilumab for asthma or NP [6]. Although excluding patients with a history of other type 2 biologic use can help ensure that changes seen in outcomes assessed are due to mepolizumab, not excluding these patients − as done in this study − supports the curation of a population of patients seen in real-world clinical practice whereby treatment decisions often involve switching therapies. The claims-based database study also reported significant reductions in incidence of sinus surgery post-mepolizumab (43% 12-months post-mepolizumab, *p*<0.001) [6], further substantiating the effectiveness of mepolizumab and the long-term action of the treatment. It should be noted that while this other real-world study used a claims-based database, our study benefits from a wider linked EMR and claims-based registry, which enabled the identification of CRSwNP-related clinical outcomes.

Real-world studies are critical in demonstrating the true benefit of a given treatment, beyond a randomized controlled trial (RCT) setting. However, as many real-world studies use claims-based databases, the data lack necessary contextual information around the reasons behind healthcare visits, procedures and treatment decisions, as well as cause of symptoms. The OM1 RWDC used in this study, is a regularly updated, multi-source dataset, derived for a range of clinical networks, including an enhanced ear, nose and throat physician routine practice registry, allowing for integration of detailed clinical information from EMR, capturing nuanced patient symptoms, comorbidities, and treatment patterns that are often missed in traditional claims-only databases. Unlike this study, in common with most RCTs, in the SYNAPSE trial [5], patients were younger (48.6 years), the majority were male (139/206, 67%) and the patient population was less diverse (SYNAPSE, 6/206, 3% Black or African American vs. this study, 30/205, 12%, respectively). It is evident that RWE better reflects a population seen in clinical practice and allows a higher level of generalizability, which is reassuring for clinicians when it comes to decision-making with mepolizumab.

The following limitations should be considered. Firstly, it is important to note that patterns of HCRU are driven by many factors, some of which are unrelated to the patient’s clinical status (e.g., routine appointments). As such, changes in HCRU are only a surrogate of treatment benefit and the current analysis did not distinguish the different reasons for HCRU changes. Additionally, the pre-/post-study design does not fully control for temporal changes. Factors that may vary over time are not accounted for in the post-mepolizumab period, meaning that some observed differences could be attributable to influences other than mepolizumab treatment. The absence of a comparator control group not receiving mepolizumab also limits the ability to fully exclude regression to the mean as a contributor to the reductions observed. However, the statistically significant and consistent improvements across multiple outcomes, supportive sensitivity analyses and the alignment of these results with those from the SYNAPSE trial [5] make this an unlikely explanation for the changes seen.

Secondly, symptoms were extracted from clinical notes and only report a binary yes or no result for the presence of symptoms and not a qualitative assessment of the improvement or worsening of symptoms. To gain further understanding of the effect of mepolizumab on symptoms, future investigations could also analyze the improvement or worsening of symptoms to gauge the extent of symptomatic change given patients may experience substantially less symptoms with mepolizumab [22], and this type of improvement was not captured within this analysis. While including patients with asthma enhances the representativeness of the patient population to reflect real-world clinical practice, the treatment effects seen could be secondary to improvements in asthma symptom control. Furthermore, the indication for individual prescriptions was not consistently recorded in this dataset, limiting our ability to distinguish medications prescribed specifically for asthma from those prescribed for CRSwNP. Yet, reductions were similar between the overall population and population of patients without comorbid severe asthma (sensitivity analysis), demonstrating that CRSwNP-related improvements were not exclusive to patients with comorbid severe asthma, or necessarily secondary to asthma symptom control.

While the sample size was relatively small, it was still sufficient to power the study based on a minimum requirement of 174 patients to achieve 80% power. In a larger study differences observed between treatments are more likely to achieve significance, therefore our results are reassuring. Lastly, despite the study follow-up period being in line with the time period recommended by EUFOREA for evaluating biologic treatment effectiveness [20], the 6-month follow-up period does not provide insight into the longer-term outcomes of treatment and future investigations with long-term follow-up should be explored. Additionally, although the inclusion criteria required only a single recorded dose of mepolizumab, which limited our ability to determine long-term treatment continuation, a sensitivity analysis was conducted which required at least two recorded administrations, and the results showed similar patterns of improvement with larger effect sizes. Nevertheless, adherence and full treatment-duration data were not assessed, which represents a limitation of the study. Although benefits are expected to be maintained with longer-term mepolizumab use [5], future real-world studies in patients with CRSwNP are warranted to further demonstrate this.

In conclusion, in a real-world setting mepolizumab is associated with reductions in HCRU and improvements in clinical outcomes among patients with CRSwNP, substantiating RCT findings in a broader patient population. In addition to alleviating HCRU burden, mepolizumab demonstrates the potential to alleviate medication burden in patients with CRSwNP. This study offers real-world insight that may support clinician decision-making with mepolizumab in this patient population.

## Supplementary Information

Below is the link to the electronic supplementary material.

Supplementary Material 1.

## Data Availability

Data are owned by OM1 and accessed by GSK to address the prespecified research questions only. The data are available via OM1 data license.
