# Supplementary material for: Mepolizumab reduces healthcare resource utilization in patients with chronic rhinosinusitis with nasal polyps: a linked EMR and claims-based pre-post study
Source: Allergy Asthma Clin Immunol. 2026 Jun 21;22:50. doi: 10.1186/s13223-026-01038-w (PMC13602526; doi:10.1186/s13223-026-01038-w)
Supplement: Supplementary file 1 [file 13223_2026_1038_MOESM1_ESM.docx]

**Supplementary Material**

***Supplementary Figure 1: Study design***

***Supplementary Figure 2: Patient attrition***

*Date of first claim or prescription defined as the index date.

CRS, chronic rhinosinusitis; NP, nasal polyps.

***Supplementary Figure 3: CRSwNP-related HCRU, pre- and post-mepolizumab, in patients with
≥2 mepolizumab records***

*Changes in CRSwNP-related HCRU were assessed by mean difference (95% CI) in the number of outpatient, specialist and ED visits 6-months pre- versus 6-months post-mepolizumab initiation.

CI, confidence interval; CRSwNP, chronic rhinosinusitis with nasal polyps; ED, emergency department; HCRU, healthcare resource utilization; mepo, mepolizumab; SD, standard deviation.

***Supplementary Figure 4: CRSwNP-related procedures, concomitant medications and signs
and symptoms, pre- and post-mepolizumab, in patients with CRSwNP without concomitant
severe asthma***

*****INCS refers to prescriptions only and does not include over‑the‑counter medications; **^✝^**Changes in clinical outcomes were assessed by mean difference (95% CI) in the number of diagnostic and surgical procedures, medication records, signs and symptom mentions and asthma-related medication prescription records 6-months pre- versus 6-months post-mepolizumab initiation.

CI, confidence interval; CRSwNP, chronic rhinosinusitis with nasal polyps; CT, computed tomography; ESS, endoscopic sinus surgery; INCS, intranasal corticosteroid; mepo, mepolizumab; MRI, magnetic resonance imaging; OCS, oral corticosteroid; SD, standard deviation.

*Supplementary Table 1: CRSwNP-related HCRU, procedures, concomitant medications and signs and* ***symptoms, pre- and post-mepolizumab***

|  | **Proportion of patients, n (%)** | | | **Percent reduction, %  (6-months  pre- vs post-mepolizumab** |
| --- | --- | --- | --- | --- |
| **Outcomes** | **12-months pre-mepolizumab period (N=245)** | **6-months pre-mepolizumab period (N=245)** | **6-months post-mepolizumab period (N=245)** |  |
| **CRSwNP-related HCRU visits** | | | | |
| Outpatient | 243 (99) | 225 (92) | 179 (73) | -21% |
| Otolaryngologist | 129 (53) | 115 (47) | 92 (38) | -19% |
| Allergist | 80 (33) | 73 (30) | 49 (20) | -33% |
| Pulmonologist | 23 (9) | 20 (8) | 14 (6) | -25% |
| ED | 6 (2) | 3 (1) | 2 (1) | 0 |
| **CRSwNP-related procedures** | | | | |
| Sinus debridement | 43 (18) | 34 (14) | 13 (5) | -64% |
| ESS | 36 (15) | 23 (9) | 13 (5) | -44% |
| Nasal endoscopy | 182 (74) | 152 (62) | 115 (47) | -24% |
| Sinus CT | 66 (27) | 46 (19) | 7 (3) | -84% |
| Polypectomy | 4 (2) | 3 (1) | 2 (1) | 0 |
| **CRSwNP-related concomitant medications** | | | | |
| OCS | 124 (51) | 89 (36) | 65 (27) | -25% |
| Oral antibiotics | 121 (49) | 94 (38) | 57 (23) | -39% |
| INCS | 79 (32) | 65 (27) | 50 (20) | -26% |
| **CRSwNP-related signs and symptoms** | | | | |
| Nasal obstruction | 122 (50) | 103 (42) | 74 (30) | -29% |
| Nasal discharge | 106 (43) | 90 (37) | 62 (25) | -32% |
| Facial pain/pressure | 57 (23) | 48 (20) | 32 (13) | -35% |
| Anosmia | 46 (19) | 37 (15) | 16 (7) | -53% |

CRSwNP, chronic rhinosinusitis with nasal polyps; CT, computed tomography; ED, emergency department; ESS, endoscopic sinus surgery; HCRU, healthcare resource utilization; INCS, intranasal corticosteroid; OCS, oral corticosteroid
